# Inter-rater reliability of Glasgow Coma Scale at a Referral Care Facility in Uganda: effect of an educational intervention among health workers

**DOI:** 10.4314/ahs.v25i3.13

**Published:** 2025-09

**Authors:** Peter Nyakuni, Betty Kinkuhaire, Diana Mbatudde, Laura Brennaman

**Affiliations:** 1 Muni University, Nursing; 2 Mbarara University of Science and Technology; 3 The Aga Khan University - Uganda, Nursing and Midwifery; 4 Nova Southeastern University, Ron and Kathy Assaf College of Nursing; 5 Mbarara University of Science and Technology, Department of Nursing

**Keywords:** Glasgow Coma Scale, Professional Knowledge, Inter-rater reliability

## Abstract

**Background:**

The Glasgow Coma Scale is a widely used tool in the assessment and tracking of patients with acute head and brain insults. However, its reliability and application in low-resource clinical settings have been least explored.

**Purpose:**

The study aimed at determining the IRR of GCS among health workers and evaluating the effect of a standardized GCS Aid as an educational intervention in improving the accuracy and IRR of the GCS when assessing patients with neurological deficits at a tertiary facility in Uganda.

**Methods and Materials:**

The study employed a pre-test, post-test quasi-experimental design. Participants included health workers working within wards with adult patients with acute brain insults. A total of 45 participants completed the study.

**Results:**

Findings indicate that higher training levels increased knowledge of GCS (F = 3.753, p = 0.01). The GCS AID improved the accuracy and IRR of the GCS post-test with eye-opening showing the greatest improvement (α, 0.65-0.88) followed by verbal (0.74 to 0.89), motor (0.83 to 0.93).

**Conclusion:**

The use of the GCS-AID improved the IRR of GCS among health workers in the assessment of patients with acute brain insults.

## Introduction

Since its inception in 1974, the Glasgow Coma Scale (GCS) has become a hallmark for clinical assessment of the neurological status of patients with acute brain injury 1. Developed as a primary tool to aid the triaging of the severity and recovery of patients with traumatic brain injuries in speciality care settings 2, 3, currently, the GCS is extensively used in different hospital care settings, including general wards, high dependency units and intensive care units, as a key tool for assessing and monitoring recovery for patients with altered levels of consciousness from different disease states. Several studies have determined the ability of the GCS to predict mortality in many mortality-predictive models for different patient populations. For example, a study by Tran et al. 4 at Mulago National Referral Hospital in Uganda demonstrated that a low GCS on admission among patients with traumatic brain injury was significantly associated with poorer patient outcomes. Related studies by various other authors have yielded similar results 5-7, 8, 9, 10, 11, 12, 13, 14. Key to the ability of the GCS to determine the severity of acute brain injury, guide patient care, and predict pa- patient outcomes; the GCS should be applied reliably, accurately, and consistently as a score 15. The accuracy of the GCS can only be maximised if all three components (i.e., motor response, verbal response, and eye-opening) of the scale are reliably assessed each time the score is recorded by any of the members of the healthcare team. To take these scores accurately, all members of the healthcare team should be knowledgeable about the GCS components and scoring systems. However, wide disparities in the application by different health workers have been documented. Some studies have es-established that the knowledge of the components of GCS is affected by the area of speciality, and time from graduation among nurses and or physicians^3^, ^15^-^18^. Other studies have also indicated that the limitation of knowledge regarding GCS affects the skill of application and reliability of the score,^19^, ^20^, thus affecting patient care and outcomes. These discrepancies warrant that clinical teams involved in the assessment and documentation of the GCS are constantly updated on the techniques for GCS scoring in different care settings, where it is commonly applied 1. With the resource limitations of low-income countries, including severe constraints in the availability of computerised tomography scanning, plain radiology, and other imaging studies, the use of GCS in the care of patients with altered consciousness levels provides even more important clinical information. However, the accuracy in the assessment of neurological status using the GCS has been less studied in these resource-limited settings despite its widespread use in such settings. The techniques used for assessing pa- patients to determine GCS have changed over the years. Over the last 40 years, since its inception into clinical practice in 1974, the Glasgow Coma scale has evolved and currently involves evidence-based techniques that have been shown to improve its accuracy in clinical application. The most current and currently used guidelines of the GCS are summarised into a quality improvement tool called the GCS Aid, developed by the Institute of Neurological Sciences, NHS Greater Glasgow, and Clyde. The GCS Aid explains recent evidence-based techniques that should be adhered to during bedside application of the GCS. For example, there have been changes in how to elicit safe and appropriate use of painful stimuli from the traditional sternal rub to applying pressure to the peri-orbital region and the nail bed. Additionally, recognition of lateralizing signs and determination of pupillary size and reflexes are considered essential^21^. Despite these developments, our research team noticed that many health workers in Mbarara Regional Referral Hospital still apply the sternal rub to elicit pain stimuli for eye-opening and motor responses, with wide variations in scores assigned to one particular patient within the same time frame. To date, no study has evaluated the level of knowledge and accuracy of GCS by health workers in Uganda and whether the healthcare workers are up to date with the techniques and methods of GCS scoring. The purpose of this study was, therefore, to determine the accuracy and inter-rater reliability of the GCS among health workers and also establish the effect of an educational intervention on the inter-rater reliability of the GCS among health workers (doctors and nurses) in four different wards settings (Emergency departments, surgi- cal wards, medical wards, and ICU) of Mbarara Regional Referral Hospital. The specific objectives of the study were to assess the level of knowledge of health workers regarding the GCS; to determine the accuracy and consistency of GCS scores among health workers; and to evaluate the effect of an educational intervention (GCS AID) on the accuracy and consistency of the GCS scores. Understanding these parameters may be essential in deciding if continuous professional courses are required to improve the application of GCS in the clinical management of patients with acute brain injury in Uganda.

## Materials and Methods

### Study design, settings, and population

The study employed a single-group pre-test post-test quasi-experimental design. The intervention in the study involved training participants on GCS assessment using the GCS AID, and afterwards assessing the accuracy of scores on patients with acute brain insults. The study sites included four units of Mbarara Regional Referral Hospital (MRRH), which receives patients with neurological deficits requiring GCS application in assessment. The hospital has two departments that handle patients with neurological disorders, i.e., the neurology division in the department of Internal Medicine and the neurosurgery division in the department of Surgery. From these divisions, we selected patients for the hospital unit, i.e. the emergency department, surgical ward, medical ward, and Intensive Care Unit. MRRH has a catchment population of over four million people from Mbarara, Bushenyi, Kiruhura, Ibanda, Buhweju, Rubirizi, Mitooma, Isingiro, Ntungamo, and Sheema districts. The study population involved doctors and nurses working in the included units who are directly involved with the primary assessment of patients with neu- neurological deficits. Patients managed within the various units were admitted with a wide range of disease conditions, including stroke, encephalitis, meningitis, post-brain surgery, and traumatic brain injury.

Sample size and recruitment of participants. Participants were recruited using convenience sampling techniques. Any physician and nurse working at the study site who happened to be present during the time of the study were selected until the required sample size was attained. The ratio of the population of doctors to nurses in all study sites was 2:1. An a priori power analysis using a margin of error, α =.05 and power β =.80, indicated that the study was powered to detect a large effect with a total sample size of 58. However, 51 participants participated in the pre-intervention phase, and 45 completed all the phases of the study. Doctors included consultants, physicians, residents, general practitioners, and intern doctors; while nurses with a master's degree in critical care nursing, bachelor's in nursing, diploma, and enrolled general nursing cadres. Student nurses who were upgrading but licensed to practice at their previous level of training were also included in the study. Nursing and medical students were excluded from the study.

### Data collection methods and tools

The data collection process comprised five key phases, i.e., patient and health worker identification; pre-intervention participant observation; knowledge assessment; GCS AID training, and post-intervention participant observation. In the first phase, i.e. patient and health worker identification, patients with neurological deficits and altered levels of consciousness whose GCS ranged from four to fourteen were identified by the researcher on different wards.

The patients selected were those who were stable without a rapidly changing neurological status (stable GCS within the past 24 hours) to ensure the stability of the assessment. Following this, the principal investigator (PI) and the research assistant (RA) obtained the GCS score for that patient for the identified patients.

The scores of the PI and RA served as the reference standard for the study participants. Both the PI and the RA had to get the same scores before it was considered absolute. The PI was a student finalising his master's in critical care nursing, and the research assistant was a neurological nurse. In the second phase, the ‘pre-intervention observation’, the PI and RA introduced themselves to the participating- ing health workers and informed them that there was an ongoing quality improvement study, but were not told the specific purpose of the study. Three participants (raters) were then asked to score the same patient independently without observing the other. Each of the 51 participants were observed independently, noting compliance with the steps recommended in the GCS AID. We recorded the various steps each participant made in the assessment (The correct sequence of performing GCS is outlined in Box 1 below). Afterwards, the par- participants assigned a score for each of the components of the GCS and computed a final score for the patient. Their scores were compared among each of the three participants and with the gold standard (PI and RA), who had initially scored the same patient. Each of these scores was entered into a checklist, where the specific GCS scores of eye-opening, verbal response, and motor response were recorded. This process was repeated until the required sample size and the whole data collection process lasted one week. A total of 15 patients were identified and used for the pre-intervention observation.

The third phase involved a pre-intervention knowledge assessment. After the pre-intervention observation, participants were requested to fill out a structured questionnaire comprising 16 questions regarding GCS knowledge. Given that the pre-intervention knowledge assessment lasted for one week, the researcher requested participants not to share the information within that week to minimise the information diffusion effect. Data from these questions were then analysed. In the fourth phase, “the GCS training intervention,” participants underwent GCS training using the GCS AID. The training involved both knowledge and skills components for performing a GCS. The training comprised the entire group of participants and was facilitated by the PI and research assistant. The training session took place on a single day and lasted about 2 hours. In the fifth phase, the post-intervention participant observation following training, the same participants who participated in the pre-test observation and knowledge assessment were requested to conduct GCS on a fresh set of patients. The observation process was conducted in the same way as in the pre-intervention- tion participant observation. Equally, a total of 17 patients were used for the post-intervention observations. Two data collection tools were used throughout the data collection process, i.e. the pre- and post-intervention- tion observation checklist and a structured questionnaire for pre-intervention knowledge assessment. Both tools are validated tools used with permission from Graham Teasdale et al. (2014) accompanying the GCS AID training manual. To ensure the validity of the tools in our study settings, the researcher piloted the observation checklist among 6 participants at Lira Regional Referral Hospital, about 500km away from MRRH, but with similar settings and services. No items were removed or modified from both tools after piloting.

### Data Management and Analysis

Researchers kept all the data for each participant together until analysis by writing the subject code number on each page of each checklist. Data were entered and stored in Microsoft Excel. We used STATA version 11.2 software for data analysis. We entered the scores that each rater assigned for each patient evaluation for every component of GCS and compared the participants' rated scores to the gold standard scores assigned by the study (PI & RA) to determine the accuracy of the GCS ratings. Two-way ANOVA was used to compare the mean knowledge of GCS between the healthcare workers with different training levels, working in different wards, and with different working experiences. Pearson's Chi-Square (χ2) was used to determine the impact of educational intervention on the sequence of performing GCS and the type of stimuli used to elicit pressure. We used a paired sample t-test to evaluate the impact of an education intervention on the accuracy of performing GCS. We compared the scores assigned by study participants to each other to assess interrater reliability. Krippendorff's alpha was used to determine the extent of the agreement by raters for both the pre-intervention and post-intervention observations, and among each component of GCS. The use of Krippendorff's alpha to determine the extent of the agreement by raters is a robust statistical test that can handle missing values, given that each observation was assessed by at least two raters.

### Ethical Considerations

We obtained ethical approval from the Research Ethics Committee of Mbarara University of Science and Technology (MUST-REC) with approval number MUREC 03/12-17. During the data collection process, participants' autonomy was ensured by ensuring all participants signed a consent to participate in the study following an explanation of the purpose of the study. Consent was also obtained from patients' families or legal caretakers from whom the GCS was performed. We also ensured that no more than three participants obtained the GCS on a particular patient. Participant codes were used to identify participants without the use of any personally identifying information. All participant data has been kept under lock and key by the PI. The training participants were given some allowance which catered for their transport refund and refreshments during the training.

## Results

Out of the 58 anticipated participants, we recruited 51 participants in the pre-intervention phase from the emergency department, medical, ICU, and surgical wards, yielding a response rate of 87.9 per cent. Forty-five par- participants completed all five phases, giving us a completion rate of 88.23 per cent. Those who failed to complete out of the 51 recruited in the pre-intervention phase of the study were two consultants and four intern doctors. The intern doctors rotate routinely in different wards, and by the time of the post-intervention data collection, some had been transferred to other wards where patients with neurological deficits are not admitted. Participants in the study comprised doctors (n = 29, 57%), followed by nurses (n = 22, 43%). The majority of doctors were residents (n = 18, 62%), and the majority of nurses (n=20, 91 %) were general nurses. Most participants worked in surgical wards and had at least 5 years of working experience (See Table 1; socio-demographic characteristics of participants).

**Table 1:** Socio-demographic characteristics of participants

Socio-demographic variables	Frequency(*N* = 51)	Percentage
**Training Level**		
Certificate	3	5.9
Diploma	12	23.5
Bachelor	14	27.5
Resident doctors	18	35.3
Master's degree	4	7.8
**Cadre**		
General nurse	20	39.2
Critical care nurse	2	3.9
Intern doctor	8	15.7
Medical officer	1	2
Resident doctors	18	35.3
Consultant surgery	2	3.9
**Area of practice**		
Accident and emergency	20	39.2
Surgical ward	22	43.1
Medical ward	3	5.9
Intensive Care Unit	6	11.8
**Experience in years**		
1-4	24	47
5-9	15	29.4
10-14	6	11.8
15 and above	6	11.8

### Participants' Pre-Intervention Knowledge regarding the Glasgow Coma Scale (GCS)

Results from the knowledge assessment, with a possible range of scores from 0 to 16, the highest total score achieved was 15, and the lowest total score was 6. Highlights from the individual items on the knowledge assessment revealed that all the respondents (n = 51, 100%) knew the three components of the Glasgow Coma Scale (GCS, whereas six participants (11.76%) knew that ap- plying a trapezius pinch is the most correct stimulus when assessing the motor component of a Glasgow Coma Scale if the patient is unable to obey commands but localizes pain when their fingernail bed is stimulated.

### GCS, Knowledge scores based on level of training, area of practice, and work

Normality testing of the total score showed a slight skew to the right. We, therefore, performed a two-way ANOVA to compare the effect of training level, area of practice, and years of working experience on the mean GCS scores. Table 3 shows the results of the analysis. We excluded speciality from the analysis because some cells had fewer than five individuals. Two-way ANOVA showed that there was a statistically significant difference-between training and mean GCS Knowledge scores (F=3.753, P=.01), with the mean score increasing as the level of training increases from an enrolled certificate to a master's degree. Though there were some differences in the mean GCS knowledge scores based on the area of practice and experience in years, these did not show any statistical significance. We then performed a post hoc comparison between the level of training and mean GCS knowledge scores to identify which levels are significantly different from each other (see Table 3).

**Table 3:** GCS, Knowledge scores based on level of training, area of practice, and work experience, (n=51)

Variable	n(%)	Mean (S.E)	95% CI	*F*	*p*
**Training level**					
Certificate	3 (5.9)	10.333 (0.947)	8.412 - 12.255		
Diploma	12 (23.5)	10.417 (0.474)	9.456 - 11.377		
Bachelor	14 (27.5)	9.833 (0.641)	8.533 - 11.134	3.753	0.01
Resident doctors	18 (35.3)	12.701 (0.397)	11.897 - 13.506		
Masters	4 (7.8)	13.5 (0.947)	11.579 - 15.421		
**Area of practice**					
Emergency	20 (39.2)	12.347 (0.576)	11.178-13.514		
Surgery	22 (43.1)	11.939 (0.483)	10.414 - 12.372	0.616	0.608
Medicine	3 (5.9)	11.000 (1.063)	8.845 - 13.155		
ICU	6 (11.8)	10.250 (0.751)	8.726 - 11.771		
**Working Experience (years)**					
1 – 4	24 (47)	11.386 (0.508)	10.356 - 12.417		
5 – 9	15 (29.4)	10.371 (0.550)	9.256 - 11.487	1.010	0.468
10 - 14	6 (11.8)	11.000 (0.749)	9.481 - 12.519		
15 and greater	6 (11.8)	11.500 (0.670)	10.141- 12.859		

*Table 3: Participants' pre-intervention Knowledge scores based on the level of training*

### Effect of standardised educational intervention on the Performance, accuracy, and inter-rater reliability for GCS

Following the pre-intervention knowledge and accuracy assessment, we instituted the educational intervention. Reported here is the effect of the educational- al intervention on the accuracy (sequence, and type of stimulus) and inter-rater reliability of GCS performance post-intervention. Post-intervention, we conducted three blinded observations on a total of 15 patients.

### Effect of the educational intervention on the sequence of performing GCS

Each of the participants was observed for the sequence followed when performing the GCS based on the recommended steps. Herein, the steps are referred to as checking, observing, stimulating, and rating the patient. (See Table 4 below). The majority, 78 per cent (n = 40) participants performed initial “checking the patient”. All the participants (n = 51) followed the rest of the steps of observing the patient for eye-opening and content of speech and movements. We used a Chi-Square analysis to determine whether the proportional differences in the sequence of GCS performance pre- and post-intervention are statistically significant. Because the “checking patient step” was the only step that was poorly performed pre-intervention, it was the only step for the chi-square test to be performed. The improvement in “check the patient” represents a statistically significant improvement in the performance of the first step of the GCS, χ2(1) = 4.43, p = .035, (see Table 4 below). Pre-intervention, participants used various stimuli to elicit pressure or pain, but predominantly, participants used the sternal rub (53% of the time), and the least used stimulus was periorbital pressure. There was a statistically significant change in the type of pressure stimuli used from using sternal rubs pre-intervention to using periorbital and nail bed press post-intervention.

**Table 4:** Effect of the GCS Aid on the sequence and type of stimulus used when performing GCS

		Phase			χ^2^	*p*
**Sequence for GCS**		**Pre-intervention** **(*n* = 51) %**	**Post-intervention** **(*n* = 46)%**	**Total**		
Patient	Performed	51(100)				
Observation	Not performed	0				
Patient	Performed	51(100)				
Stimulation	Not performed	0				
Patient	Performed	51(100)				
Scoring	Not Performed	0				
Check the	Performed	40 (78.4)	43 (93.5)	83		
patient	Not performed	11 (21.6)	3 (6.5)	14	4.434	*0.035
**Type of Pain Stimulus**						
Sternal rub		27 (53)	2 (4)	29		
Nail bed pressure		9 (17)	31(67)	40	36.29	≤0.01
Periorbital pressure		7 (14)	10 (22)	17		
Trapezius pinch		8 (16)	3 (7)	11		

*Table 4: Effect of the Educational Intervention on the Sequence of Performing the GCS.*

### Effect of the Educational Intervention (GCS Aid) on the Accuracy of GCS Scores

We computed a mean score for each component of the GCS for all 51 observations pre-intervention. We compared it to the mean score of the gold standard for each of the GCS components, as well as the total score, from which we computed the percentage variation (See Table 5). The mean differences between participants' scores and the gold standards reduced from the pre-intervention assessments to the post-intervention assessments in each of the three components of the GCS and the total score indicated- ed an improvement in the accuracy of each component and total GCS scores. However, only the eye-opening and verbal response score components showed statistically significant differences between the pre-intervention and post-intervention scores (p = .025 and .048, respectively).

**Table 5:** Effect of the GCS Aid on the accuracy of GCS by different raters

	Pre-intervention			Post-intervention				
	Gold Standard *m*(*SD*) (*n* = 17)	Raters' *m*(*SD*) (*n* = 51)	% variation	Gold Standard *m*(*SD*) (*n* = 15)	Raters' *m*(*SD*) (*n* = 46)	% variation	*t*	*p*
Eye opening	2.76(1.66)	2.55(1.03)	7.80	2.13(1.45)	2.11(.78)	1.04	2.313	.025
Verbal	2.18(1.47)	2.12(.90)	2.70	2.53(1.59)	2.44 (.97)	3.51	-2.031	.048
Motor	3.18(1.78)	3.16(1.38)	0.62	3.43(1.85)	3.36(1.05)	2.08	-.275	.785
Total score	8.12(2.85)	7.75(2.78)	4.59	7.93	7.96(2.21)	0.28	-.510	.612

*Table 5: Effect of the Educational Intervention on the Accuracy of GCS Scores.*

### Effect of GCS AID educational intervention on inter-rater reliability (IRR) of the GCS

We calculated the Interrater reliability (IRR) using Krippendorff's alpha to determine the internal consistency between GCS assessments performed by the different healthcare workers. The reliability of Krippendorff's alpha ranges from 0 (no agreement) to 1 (perfect agreement). To identify potential areas with high or low reliability, the consistency of each of the three components of GCS was analysed separately, and then we also computed the IRR for the total score. All three components showed a marked improvement in Krippendorf's alpha, with the post-intervention values being at least 0.8. Eye-opening showed the least IRR but overall showed the largest improvement in the IRR from the α = .65 pre-intervention to α = .88 post-intervention. (See Table 6 below).

**Table 6:** Effect of the educational intervention on inter-rater reliability of GCS

Component	Inter-rater reliability (IRR)	
	**Reliability (Pre-intervention)**	**Post-intervention**
Eye	0.65	0.88
Verbal	0.73	0.89
Motor	0.87	0.93
Total	0.90	0.92

*Table 6: Effect of the Educational Intervention on the IRR.*

α – Krippendorff's alpha for inter-rater reliability

## Discussion

The purpose of this study was to determine the inter-rater reliability of the Glasgow Coma Scale and examine the effect of GCS Aid as an educational intervention on the healthcare workers' inter-rater reliability of the GCS on patients with neurological deficits, with the role of facilitating better assessments and aiding well-informed clinical decisions. The results of this study indicate a wide variation of health workers' knowledge about the GCS coma scales, from as low as 11.67% to as high as 100% in stating the three components of GCS. Most of the poorly performed scores on the knowledge assessment relate to questions on clinical scenarios requiring the application of the basic knowledge of GCS, types of stimuli to be used, and scoring if the patient is oriented to assess confusion. The results also show that the level of training of health workers directly affects the knowledge scores, with a higher level of training being associated with a higher score on the pre-intervention knowledge assessment. Our study found that resident doctors (post-graduate students in medicine or surgery) had higher mean scores of knowledge compared to healthcare workers with certificate levels of training, implying that the level of training greatly influences the knowledge of GCS. The area of practice and years of working experience did not show a statistically significant difference in the knowledge score for the GCS scale. Not-withstanding, most of the participants in our study had a working experience of 5 years or more. Further, overall the results also indicate the positive effect of the GCS Aid in improving the sequence of GCS performance, using the correct type of stimuli, and improving the accuracy and inter-rater reliability of the GCS assessments.

The findings of this study in relation to the pre-intervention GCS knowledge are, in part, consistent with several similar studies. A study among nurses in Singapore 15 and among nurses in Vietnam 22 showed a great disparity in the knowledge of the GCS between nurses of different demographics, especially regarding clinical scenarios requiring the application of the basic knowledge. Similar studies are reported among physicians 17. However, these studies found a statistically significant difference between the area of practice and years of experience, for example, physicians who demonstrated a poor knowledge level and recall, especially after spending many years after graduation (more working experience). Our study only showed a statistically significant difference in the knowledge level in regards to the level of training, especially between resident doctors and master's level trained professionals and diploma nurses. Our study sample did not yield a statistically significant difference regarding the area of practice or speciality and years of working experience. This difference might be attributable to the limited emphasis given to training for diploma nurses while in nurse training schools. Also, it is anticipated that as one progresses through professional medical education, one accumulates a wider knowledge and understanding of concepts.

This disagrees with studies in Jordan 23 and Nigeria 17, whose findings showed no significant relationships between educational level and knowledge of GCS in their correlation study. The findings in our study bear more implications in Uganda because the majority of nurses hold certificates and diploma levels of training in nursing, and they occupy the largest proportion of the workforce in the country. Because every healthcare worker involved in the care of critically ill patients should have adequate knowledge and skill in GCS to provide good communication and triage, this warrants the need for continuous education (CE) on GCS assessment in this category of health professionals in key clinical areas where the tool is regularly applied. Contrariwise, the years of working experience and area of practice did not appear to affect the knowledge of GCS. This signifies the role of CMEs in updating the skills and knowledge of all clinical practitioners. Our findings extend previous research by Al-Quraan & Eid AbuRu in Jordan, which showed no relationship between years of experience and knowledge of GCS in their correlation study 23. The pre-intervention findings indicated an inconsistent sequence of taking GCS, the correct type of stimulation, and accuracy consistent with several other studies. These findings further extend a survey by Reith et al. (2014), which demonstrated substantial variation in several aspects of the examination of a patient to determine their GCS, in particular, discrepancies in the type of stimulus used to provoke a response. Additionally, research by Bledsoe et al. 24 in a prospective observational American study on the accuracy of GCS scoring reported that out of a total of 2,084 GCS observations, the overall accuracy was 33.1 per cent. This difference in mean score may have been caused by the failure of the raters to follow the standardised sequence in assessing patients using GCS, as seen in our study. Regarding the pre-intervention inter-rater reliability, the study findings concur with another study by Gill et al. (2004) conducted among two resident doctors to determine the inter-rater reliability of GCS when performed on 116 patients. Gill's study showed a moderate agreement in GCS scores between the two residents. The agreement for the GCS components was 74 per cent for eye-opening, 55per cent for verbal, and 72 per cent for motor response. However, our study showed a wide variation concerning eye-opening and less with the motor response. Our study showed that the IRR was poorest for eye-opening (0.65), followed by a verbal response (0.74).

The motor response and the total score had somewhat good inter-rater reliability (0.87 and 0.9, respectively) pre-intervention. This disagrees with the study by Heron et al. (2001), where verbal response demonstrated the highest level of inter-rater reliability, whereas motor response had the lowest IRR value. This disagreement might be explained by the modifications in the GCS tool since 2001, when Heron's study was conducted. Also, the low reliability for eye-opening and verbal responses should have been caused by the failure of the raters to follow sequential steps in the assessment and using wrong methods to elicit responses using pressure stimuli, as reported earlier in the results. Additionally, although the inter-rater reliability of the total score was high, individual components of GCS provide more information about patient conditions and trends than the overall total score, and the inconsistency in the component scores may pose a risk to the accurate trending of patient conditions. The agreement for GCS or any other tool that is used in a clinical setting should be at least 0.8 between raters for the score to be reliable in the clinical application 25. The GCS AID as an education intervention demonstrated an ability to improve the inter-rater reliability between participants and the gold standard to recommended levels. Post-intervention, all components of GCS showed improvements in the inter-reliability test of 0.80 and above, the minimum for any tool to be acceptably reliable. These findings are consistent with a study by Teles et al 26., who assessed the effectiveness of the self-instructional module and demonstrated an improvement in the knowledge and skills regarding the use of GCS in a hospital in Belgaum. Before the educational intervention, 84 per cent of participants had moderate skills, 15 per cent had inadequate skills, and only 2 per cent had adequate skills required to perform GCS correctly. After the intervention with the self-administered module, 36 per cent were able to perform the GCS scoring with adequate skill. The findings of this study and those from other earlier studies, like Teles' study, imply that with well-tailored educational interventions like the GCS AID, patient assessment and clinical decision-making can be improved more reliably, especially with low-cost interventions.

## Limitations

Our study involved a small convenience sample of a size of 51 participants. This might ultimately limit the generalizability of the study findings. However, the study involved a mixed group of clinical practitioners and therefore may help understand the use of the GCS tool in the different health worker demographics. Additionally, the study was carried out in a teaching hospital, and therefore, the results might not necessarily apply to non-academically affiliated hospitals. Moreover, the results may not be generalizable to other hospitals in Uganda because the greatest proportion of the respondents were resident doctors who only practice in three teaching hospitals in Uganda. Our assessments by the multiple raters were within 10 minutes apart; some of the variations could have been due to actual patient changes in function rather than inter-rater variation. However, because we chose stable patients without rapidly changing GCS within the past 24 hours, we believe this variation was limited.

## Recommendations

The authors recommend the following actions for clinical practice, education, health care administration, and research. In clinical speciality units where patients with neurological deficits are associated with alterations in their level of consciousness, continuous medical education on GCS should be implemented by hospital and unit administrators to improve the accuracy and reliability of such assessment of patients.

The techniques for GCS assessment have changed over the years, and therefore, health workers should be continuously updated on these techniques, including all cadres, areas of speciality, and working experience. This will ultimately improve the uniformity of scores, better patient case management, and decision-making, thus improving patient outcomes. To research, a larger, more robust study is warranted in different hospitals to provide a clearer understanding of the application of the GCS in different hospitals.

## Conclusion

This study demonstrated a moderate knowledge level for the utilisation of the Glasgow Coma Scale in different clinical settings and different cadres of health workers. Knowledge was poorest for types of stimuli used and the sequence of GCS scoring. The GCS knowledge pre-intervention was influenced by the level of professional training but was not affected by the area of practice and working experience. The accuracy and inter-rater reliability were poor for eye-opening followed by verbal response pre-intervention, and these were mostly affected by the lack of sequential steps in the GCS assessment. The GCS AID improved the accuracy and inter-rater reliability for the individual components and total scoring of GCS.

## Figures and Tables

**Figure 1:**
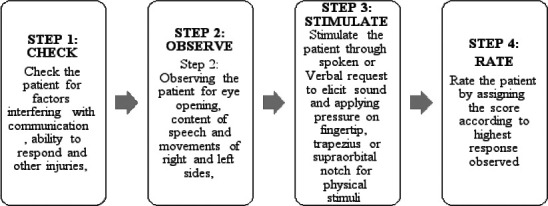
Steps in the Sequence for Performing the Glasgow Coma Scale

**Table 2:** Knowledge of participants about GCS, (n=51)

Questions	Correct Responses *n* (%)
The three components of GCS?	51(100)
Combinations of each component of GCS	50(98)
The sequence of responses assessed in the eye component	42(82.35)
The maximum score for each Glasgow Coma Scale components	25(49.01)
Steps to follow during GCS assessment	38(74.5)
The rationale for the CHECK step in the assessment Scoring a	36(70.58)
patient who is awake and looking at you	45(88.23)
Scoring a patient whose eyes are swollen and unable to open	46(90.19)
The score for a patient who answers a wrong date but correct name and place	20(39.21)
What to do for a patient who is unable to obey commands but	06(11.76)
bend their elbow when their fingernail bed is stimulated.	42(82.35)
Scoring a patient who reacts to supraorbital pressure by moving	35(68.62)
their hand up to his face.	44(86.27)
Scoring a patient with decorticate posturing	32(62.74)
How to interpret E2, V3, M5,	44(86.27)
Best response on the patient's right-hand side	39(76.47)
The lowest score on the GCS scale	
The upper limit for comatose patients	

## References

[1] Teasdale G, Maas A, Lecky F, Manley G, Stocchetti N, Murray G (2014). The Glasgow Coma Scale at 40 years: standing the test of time. The Lancet Neurology.

[2] Namiki J, Yamazaki M, Funabiki T, Hori S (2011). Inaccuracy and misjudged factors of Glasgow Coma Scale scores when assessed. Clinical Neurology and Neurosurgery.

[3] Riechers GR, Ramage A, Brown W, Kalehua A, Rhee P, Ecklund MJ (2005). Physician Knowledge of the Glasgow Coma Scale. Journal of Neurotrauma.

[4] Tran TM, Fuller TA, Kirybwire J, Mukasa J, Muhumuza M, Senyonjo H (2015). Distribution and Characteristics of Severe Traumatic Brain Injury at Mulago National. World Neurosurgery.

[5] Bastos PG, Sun X, Wagner DP, Wu AW, Knaus WA (1993). Glasgow Coma Scale score in the evaluation of outcome in the intensive care unit: findings from the Acute Physiology and Chronic Health Evaluation III study. Critical Care Medicine.

[6] Grmec š, Gašparovic V (2000). Comparison of APACHE II, MEES and Glasgow Coma Scale in patients with nontraumatic coma for prediction of mortality. Critical Care.

[7] Healey C, Osler TM, Rogers FB, Healey MA, Glance LG, Kilgo PD (2003). Improving the Glasgow Coma Scale score: motor score alone is a better predictor. Journal of Trauma and Acute Care Surgery.

[8] Kondo Y, Abe T, Kohshi K, Tokuda Y, Cook EF, Kukita I (2011). Revised trauma scoring system to predict in-hospital mortality in the emergency department: Glasgow Coma Scale, Age, and Systolic Blood Pressure score. Critical Care.

[9] Mena JH, Sanchez AI, Rubiano AM, Peitzman AB, Sperry JL, Gutierrez MI (2011). Effect of the modified Glasgow Coma Scale score criteria for mild traumatic brain injury on mortality prediction: comparing classic and modified Glasgow Coma Scale score model scores of 13. The Journal of Trauma.

[10] Sartorius D, Le Manach Y, David J-S, Rancurel E, Smail N, Thicoïpé M (2010). Mechanism, glasgow coma scale, age, and arterial pressure (MGAP): a new simple prehospital triage score to predict mortality in trauma patients. Critical care medicine.

[11] Singh B, Murad MH, Prokop LJ, Erwin PJ, Wang Z, Mommer SK (2013). Meta-analysis of Glasgow coma scale and simplified motor score in predicting traumatic brain injury outcomes. Brain Injury.

[12] Weingarten S, Bolus R, Riedinger M, Maldonado L, Stein S, Ellrodt A (1990). The principle of parsimony: Glasgow Coma Scale score predicts mortality as well as the APACHE II score for stroke patients. Stroke.

[13] Wijdicks EF, Kramer AA, Rohs T, Hanna S, Sadaka F, O'Brien J (2015). Comparison of the Full Outline of UnResponsiveness score and the Glasgow Coma Scale in predicting mortality in critically ill patients. Critical Care Medicine.

[14] Zafonte RD, Hammond FM, Mann NR, Wood DL, Black KL, Millis SR (1996). Relationship between Glasgow coma scale and functional Outcome1. American journal of physical medicine & rehabilitation.

[15] Mattar I, Liaw SY, Chan MF (2013). A Study to Explore Nurses' Knowledge in Using the Glasgow Coma Scale in an Acute Care Hospital. Journal of neuroscience Nursing.

[16] Adeleye OA, Owolabi OM, Rabiu BT, Orimadegun EA (2012). Physicians' knowledge of the Glasgow Coma Scale in a Nigerian university hospital: is the simple GCS still too complex?. Frontiers in Neurology.

[17] Emejulu J, Nkwerem S, Ekweogwu O (2014). Assessment of physicians' knowledge of Glasgow coma score. Nigerian Journal of Clinical Practice.

[18] Santos WC, Vancini-Campanharo CR, Lopes MCBT, Okuno MFP, Batista REA (2016). Assessment of nurse's knowledge about Glasgow coma scale at a university hospital. Einstein.

[19] Gill RM, Reiley GD, Green MS (2004). Interrater reliability of GCS. Ann Emerg Med.

[20] Heron R, Davie A, Gillies R, Courtney M (2001). Interrater reliability of the Glasgow coma scale scoring among nurses in sub-specialties of critical care. Aust Crit Care.

[21] World Health Organization (2004). Essential Guidelines Trauma Care. Guideline.

[22] Thi Hien N, Chae S-M (2011). The Accuracy of Glasgow Coma Scale Knowledge and Performance among Vietnamese Nurses. Perspectives in Nursing Science.

[23] Al-Quraan H, Eid AbuRu M (2016). Assessment Of Jordanian Nurses' Knowledge To Perform Glasgow Coma Scale. European Scientific Journal.

[24] Bledsoe EB, Casey JM, Feldman J, Larry J, Diel S, Forred W (2015). Glasgow Coma Scale scoring is often inaccurate. Prehosp Disaster Med.

[25] Hallgren AK (2012). Computing Inter-Rater Reliability for Observational Data: An Overview and Tutorial. Tutor Quant Methods Psychol.

[26] Teles M, Bhupali P, Madhale M (2013). Effectiveness of Self Instructional Module on Knowledge and Skills Regarding Use of Glasgow Coma Scale in Neurological Assessment of Patients among Nurses Working in Critical Care Units of KLE Dr. Prabhakar Kore Hospital and Medical Research Centre. Belgaum Journal of Krishna Institute of Medical Sciences University.

